# Glucose oxidase-powered nanocatalytic medicine: Rewiring tumor metabolism for multimodal cancer therapy

**DOI:** 10.1016/j.mtbio.2025.102664

**Published:** 2025-12-09

**Authors:** Bin Wang, Naren Bao, Limei Wang, Jian Xiong, Danyang Li, Yutao Wang, Ghulam Md Ashraf, Xu Wang

**Affiliations:** a Department of Orthopedics, The Shengjing Hospital of China Medical University, Shenyang, Liaoning, China; b Department of Anesthesiology, First Affiliated Hospital of China Medical University, Shenyang, Liaoning, China; c Department of Obstetrics and Gynecology, Guangzhou Women and Children's Medical Center, Guangzhou Medical University, 9 Jinsui Road, Tianhe District, Guangzhou, 510623, Guangdong, China; d Department of Pharmacology, College of Pharmacy, Harbin Medical University, Harbin, Heilongjiang, 150081, China; e Department of Urology, The First Affiliated Hospital of China Medical University, Shenyang, Liaoning, China; f Department of Biomedical Sciences, College of Medicine, Gulf Medical University, Ajman, United Arab Emirates; g Department of Breast Surgery, The First Affiliated Hospital of China Medical University, Shenyang, Liaoning, China

**Keywords:** Glucose oxidase, Nanocatalytic therapy, Starvation therapy, Tumor microenvironment, Warburg effect

## Abstract

Cancer cells exhibit metabolic reprograming characterized by a preference for aerobic glycolysis (the Warburg effect), which supports their rapid proliferation while promoting immune invasion and microenvironment remodeling. Glucose oxidase (GOX) has emerged as a powerful therapeutic agent that exploits this metabolic vulnerability through targeted glucose depletion. Recent advances in nanocarrier technology have addressed critical limitations of native GOX, including systemic toxicity, short circulation half-life, immunogenicity, and instability in physiological environments. Engineered GOX nanoconstructs demonstrate remarkable tumor-specific cytotoxicity through dual mechanisms: (1) starvation via glucose deprivation and (2) oxidative damage through reactive oxygen species generation. Importantly, GOX-mediated modulation of tumor microenvironment parameters - acidity, H_2_O_2_ concentration, and oxygen tension - creates favorable conditions for combination therapies. This catalytic synergy enhances the efficacy of diverse treatment modalities including chemotherapy, chemodynamic therapy, photodynamic therapy, photothermal therapy, gas therapy, and immunotherapy. This review comprehensively examines recent progress in GOX-based nanocatalytic platforms, focusing on their design principles, therapeutic mechanisms, and potential for multimodal cancer treatment.

## Graphical abstract

**Figure:**
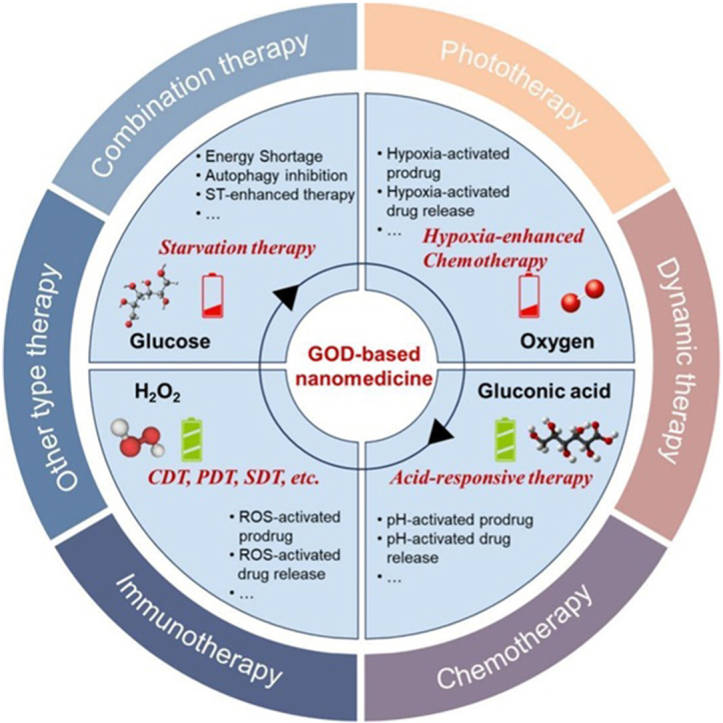

## 1 Introduction

Cancer cells exhibit a profound metabolic reprogramming known as the Warburg effect, preferentially relying on glycolysis for energy production and biosynthetic precursors rather than oxidative phosphorylation (OXPHOS), even under oxygen-rich conditions [1,2]. This adaptive metabolic shift enables tumor cells to maintain rapid proliferation despite nutrient scarcity and hypoxic microenvironments [1], while simultaneously supporting key oncogenic processes including immune evasion and metastatic potential. Beyond fueling proliferation, the Warburg effect confers multiple survival advantages that collectively promote tumor progression. The resulting lactate accumulation acidifies the tumor microenvironment (TME) [3], creating conditions that enhance tumor invasiveness while polarizing tumor-associated macrophages (TAMs) toward an immunosuppressive M2 phenotype [4]. This metabolic reprogramming simultaneously starves tumor-infiltrating lymphocytes (TILs) of glucose, further compromising anti-tumor immunity [5,6]. Additionally, by reducing OXPHOS, cancer cells minimize reactive oxygen species (ROS) production, thereby evading ROS-mediated apoptosis [7].

Multiple oncogenic pathways drive this metabolic shift [8,9], and glycolytic gene signatures correlate with poor prognosis in aggressive cancers including lung adenocarcinomas and triple-negative breast cancer [10,11]. The metabolic addiction of tumors is striking-cancer cells exhibit 10-fold greater glucose consumption and 30-fold higher glycolytic rates compared to normal cells [12,13]. This vulnerability has spurred development of therapeutic strategies targeting glucose metabolism, with several small-molecule inhibitors of glucose transporters and glycolytic enzymes currently in clinical evaluation [14,15].

Glucose deprivation exerts multi-faceted anti-tumor effects: (1) ATP depletion arrests proliferation and triggers apoptosis [16]; (2) reduced activity of ATP-dependent drug efflux pumps reverses chemoresistance [17]; (3) mitochondrial dysfunction increases ROS to lethal levels [18,19]; and (4) glucose redistribution to TILs may restore anti-tumor immunity [12]. These mechanisms collectively validate metabolic targeting as a promising therapeutic approach. Glucose oxidase (GOX), a flavin adenine dinucleotide (FAD)-dependent oxidoreductases, catalyzes the oxidation of β-D-glucose to D-glucono-d-lactone and hydrogen peroxide (H_2_O_2_) in an oxygen-dependent reaction [20]. The reaction products undergo further conversion: D-glucono-δ-lactone is spontaneously hydrolyzed by lactonase to D-gluconic acid, while H_2_O_2_ can be decomposed into oxygen and water by catalase or converted to highly reactive hydroxyl radicals (·OH) through metal ion-mediated Fenton reactions [20,21]. These biochemical transformations underline GOX's therapeutic potential, as they simultaneously deplete glucose while generating cytotoxic oxidative species.

Naturally occurring in fungi (particularly *Penicillium* and *Aspergillus* species), and insects, and some bacterial, mammalian, and plant cells [20], GOX features two functionally critical domains: an FAD-binding β-sheet region and a substrate-binding domain composed of α-helices and an anti-parallel β-sheet [21]. This structure enables its remarkable catalytic efficiency and substrate specificity, properties that have been exploited for decades in industrial and biomedical applications, from glucose monitoring in diabetics management [22] to wound through localized H_2_O_2_ production [23].

The enzyme's therapeutic potential in oncology stems from three key attributes: it simultaneously starves tumors of glucose while generating oxidative stress [24,25], (2) its pH optimum (6.5) matches the acidic TME (pH 5–7) [26], and (3) its byproducts acidify the extracellular space, which can enhance drug release from pH-sensitive carriers. However, clinical translation faces challenges including rapid proteolytic degradation [27,28], short plasma half-life [24], and potential off-target effects due to ubiquitous glucose and oxygen distribution [29].

Nanocarrier encapsulation has emerged as a promising solution to these limitations. Various platforms - including nanoparticles (NPs), organic polymers, metal-organic frameworks (MOFs), and liposomes – have demonstrated improved GOX stability and targeting [24,28,29]. Notable examples include biotinylated vesicles that selectively kill cancer cells glucose deprivation [30], polydopamine (PDA)-nanoshells that protect GOX from degradation while maintaining catalytic activity in acidic conditions [31], and polymer nanogels that enhance tumor retention while reducing systemic toxicity [32]. These advances have transformed GOX from an interesting biological catalyst to a potentially powerful therapeutic agent in cancer treatment.

The catalytic action of GOX facilitates multiple complementary anticancer mechanisms that extend beyond its primary starvation effects. The oxygen consumption during glucose oxidation intensifies tumor hypoxia, creating a favorable environment for hypoxia-activated prodrugs (HAPs) - specifically designed to undergo cytotoxic activation in low-oxygen conditions characteristic of solid tumors [33]. Concurrently, GOX-generated gluconic acid significantly acidifies the TME. This pH reduction serves a dual purpose: (1) it enhances the tumor-selective release of pH-responsive chemotherapeutic agents [34], and (2) it creates optimal conditions for Fenton chemistry, where locally elevated H_2_O_2_ levels are converted into highly cytotoxic ·OH in the presence of transition metals [24,28]. These ROS not only induce direct tumor cell apoptosis [35], but also significantly potentiate various treatment modalities including: chemodynamic therapy (CDT), photothermal therapy (PTT), and photodynamic therapy (PDT) [24,28]. The resulting oxidative stress has been shown to enhance conventional cancer treatments, with studies demonstrating improved efficacy of both chemotherapy and radiotherapy in the presence of elevated ROS [36]. This multimodal action explains why GOX-mediated therapy shows promise against treatment-resistant tumors.

**Fig. 1:**
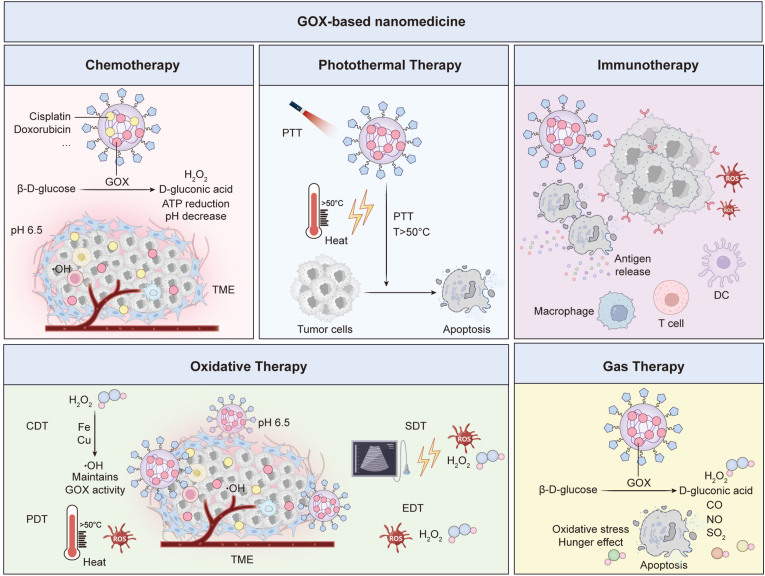
Schematic illustration of GOX-based nanotherapies. GOX induces tumor starvation through glucose depletion, and creates a conducive environment for diverse therapeutic methods, such as chemotherapy, immunotherapy, CDT, PDT, PTT, SDT, EDT, and gas therapy by modulating the acidity, H_2_O_2_ levels, and hypoxia. Adapted from Ref. 40. **Abbreviations:** GOX, glucose oxidase; CDT, chemodynamic therapy; PDT, photodynamic therapy; PTT, photothermal therapy; SDT, sonodynamic therapy; EDT, electrodynamic therapy; H_2_O_2_, hydrogen peroxide.

Clinical observations reveal strong correlations between hyperactive glucose metabolism and resistance to frontline agents like paclitaxel and 5-fluorouracil [37,38]. Similarly, the glycolytic enzyme pyruvate kinase M2 (PKM2) promotes immunotherapy resistance through immune checkpoint upregulation and recruitment of immunosuppressive cell populations [39]. These findings collectively suggest that GOX-based metabolic intervention could serve as a powerful adjunct to both traditional chemotherapy and modern immunotherapy regimens, addressing critical resistance mechanisms while simultaneously creating favorable conditions for multiple therapeutic approaches. Over the past decade, significant progress has been made in developing GOX-based nanocomposites for multimodal cancer therapy. Researchers have engineered sophisticated nanoplatforms that combine GOX with diverse therapeutic agents, including chemotherapeutic drugs, metal catalysts, enzymes, Fenton reagents, photosensitizers, photothermal agents, immune adjuvants [24,28,40]. These integrated systems demonstrate superior therapeutic outcomes compared to GOX-mediated starvation therapy alone achieving synergistic effects through simultaneous targeting of multiple cancer hallmarks (Fig. 1) [40].

Although several high-quality reviews have discussed the broader landscape of GOX-based nanotherapies, our manuscript offers a unique and timely perspective by concentrating on the design principles and mechanistic strategies that address the core challenges limiting clinical translation. Previous reviews [41] have comprehensively reported combination strategies, while a more recent review [42] provides a broad overview of catalytic nanomedicine. In contrast, our review adopts a critical, challenge-driven perspective, systematically dissecting fundamental barriers – such as the oxygen paradox, metabolic plasticity, and off-target toxicity - and highlights cutting-edge nanomaterial strategies engineered to overcome these translational limitations. We go beyond a mere descriptive summary to present a mechanism-oriented framework that critically assesses how and why advanced nanoplatforms achieve their effects, with particular emphasis on the often-overlooked but crucial roles of immunomodulation and the reversal of tumor-induced immunosuppression [203]. Accordingly, this review offers a systematic analysis of recent advances in GOX-based nanotheranostics, with particular focus on: (1) rational design principles of multifunctional nanoplatforms tailored to overcome specific biological barriers, (2) therapeutic synergies between GOX and both conventional and emerging treatment modalities, and (3) forward-looking considerations including pharmacokinetics, long-term toxicity, and scalable manufacturing. We further provide a critical assessment of current limitations and prospects of GOX-mediated cancer therapies, highlighting potential solutions and translational strategies. By emphasizing a challenge-driven design framework, this review delivers a novel and actionable perspective for the development of next-generation catalytic nanomedicines.

## 2 Mechanistic strategies and design strategies to overcome key challenges in GOX-based therapy

The therapeutic promise of GOX is tempered by a network of interconnected biological challenges that have limited its clinical translation. A thorough understanding of these barriers has guided the development of innovative nanoplatform designs. In this section, we outline the key obstacles and the rational design strategies devised to overcome them, establishing a mechanistic framework for the studies presented later in this review.

### 2.1 The oxygen paradox: self-limiting catalysis and exacerbated hypoxia

GOX's therapeutic efficacy is fundamentally constrained by its intrinsic dependence on oxygen. Within the hypoxic TME, glucose oxidation by GOX consumes O_2_, creating a negative feedback loop that can self-limit enzymatic activity [24,28]. More critically, this intensified hypoxia can activate pro-survival pathways in cancer cells, such as HIF-1α signaling, which may promote tumor invasion, metastasis, and resistance to therapy [43]. To address this challenge, several mechanistic strategies have been developed.

#### 2.1.1 Integrated oxygen economizers

The primary strategy involves co-delivering catalysts that decompose the H_2_O_2_ byproduct into O_2_. Materials such as MnO_2_ [44,45] and MOFs with catalase-like activity [46,47] facilitate a "Fenton-like" reaction: MnO_2_ + H_2_O_2_ + 2H^+^ → Mn^2+^ + 2H_2_O + O_2_, simultaneously sustaining GOX activity and generating Mn^2+^ ions for CDT, creating a self-sustaining cycle.

#### 2.1.2 External energy input

Platforms combining GOX with photothermal agents such as gold nanorods, black phosphorus, enables localized hyperthermia under near-infrared light, which enhances blood flow and oxygen perfusion while thermally accelerating GOX's kinetics [[48], [49], [50], [51]]. These design principles collectively aim to overcome the self-limiting effects of oxygen depletion and mitigate hypoxia-driven tumor progression.

### 2.2 Metabolic plasticity and tumor robustness

Cancer cells display remarkable metabolic flexibility, allowing them to survive under conditions of nutrient deprivation. Glucose deprivation can activate adaptive responses, such as autophagy, which serves as a survival mechanism [52], and the utilization of alternative nutrients - including lactate, glutamine, and fatty acids - to sustain oxidative phosphorylation [[53], [54], [55]]. This metabolic robustness significantly limits the efficacy of GOX-mediated starvation monotherapy. To counter these adaptations, nanoplatforms have been engineered to co-target compensatory pathways.

#### 2.2.1 Co-targeting adaptive pathways

Nanocarriers are engineered for the co-delivery of GOX with autophagy inhibitors, such as 3-methyladenine (3-MA), creating a "one-two punch" that enhances cancer cell death by blocking a key escape route [56].

#### 2.2.2 Multi-metabolic inhibition

Combining GOX with agents like metformin, that target mitochondrial complex I and glycolytic enzymes, thereby simultaneously multiple energy-producing pathways [12]. These strategies achieve synergistic ATP depletion and improved tumor suppression, effectively overcoming the metabolic plasticity of cancer cells.

### 2.3 Off-target toxicity and controlled activation

The ubiquitous presence of glucose and oxygen in the bloodstream poses a major challenge for GOX-based therapy, as systemic administration of free GOX can lead to severe off-target effects, including hyperglycemia and oxidative damage to healthy tissues [29]. To address this, several design strategies have been developed to achieve tumor-specific activation and minimize systemic toxicity.

#### 2.3.1 TME-responsive activation

GOX is maintained in an inert state during circulation and selectively activated within the TME using using nanocarriers that degrade under acidic conditions (e.g., ZIF-8) [57] or in response to overexpressed tumor enzymes.

#### 2.3.2 Spatiotemporal control

Advanced systems use external triggers such as near-infrared light [58] or ultrasound [59] to precisely regulate GOX release and activity at the tumor site, thereby maximizing local efficacy while minimizing systemic exposure.

#### 2.3.3 Redox homeostasis intervention

To protect normal tissues, some nanosystems incorporate redox-modulating components, such as bilirubin [60] to scavenge excess H_2_O_2_ in normoxic healthy tissues, while this protective function is suppressed in the acidic TME to allow GOX-mediated cytotoxicity. These strategies collectively enhance the safety and precision of GOX-based nanotherapies.

### 2.4 The immunological dimension: from cold to hot tumors

A major challenge in cancer therapy is the immunosuppressive TME [[205], [206]], which renders "cold" tumors largely resistant to immunotherapy. GOX-based nanotherapies offer more than direct cytotoxicity - they exert potent immunomodulatory effects. The ROS generated by GOX induce oxidative stress and immunogenic cell death (ICD), reprogramming the TME by repolarizing TAMs from the immunosuppressive M2 to the tumoricidal M1 phenotype, while also promoting dendritic cell maturation and T-cell infiltration [61,62]. These mechanistic effects position GOX-based nanoplatforms as ideal partners for combination with immune checkpoint inhibitors, effectively converting immunologically "cold" tumors into "hot" ones and enhancing therapeutic responsiveness.

This framework of challenge-driven design will be used to critically analyze the literature in the subsequent sections, moving beyond a descriptive summary to a mechanistic evaluation of the field's progress.

## 3 GOX monotherapy

GOX exerts potent anticancer effects through two complementary mechanisms: direct glucose deprivation that starves tumor cells of their primary energy source, and profound remodeling of the TME through continuous generation of H_2_O_2_ and gluconic acid. These biochemical changes collectively increase hypoxia and acidity while promoting oxidative stress through free radical free radical production, ultimately inducing tumor cell death [25]. Recent years have witnessed significant progress in developing advanced nanocarrier systems to optimize GOX-delivery and therapeutic efficacy.

Several innovative formulations have demonstrated promising results in preclinical studies. Alginate-chitosan microspheres encapsulating GOX (GOX-MS) showed marked cytotoxicity against murine breast cancer cells through H_2_O_2_-mediated lipid peroxidation damage, with subsequent *in vivo* studies confirming significant tumor growth inhibition following locoregional administration [63,64]. For post-surgical applications, researchers developed GOX-immobilized gelatin hydrogels that provided sustained enzyme release at resection sites, effectively preventing local tumor recurrence in mouse models through prolonged metabolic disruption [65]. Another promising approach utilizes engineered viral capsids as natural nanocarriers; the encapsulation of GOX within Brome mosaic virus (BMV) particles resulted in potent oxidative damage to triple-negative breast cancer cells while leveraging the inherent biocompatibility and stability of viral protein cages [66,67]. These advances in GOX nanocarrier design demonstrate remarkable potential for clinical translation by synergistically enhancing tumor starvation while precisely controlling the cytotoxic oxidative microenvironment. While these targeted strategies significantly improve tumor accumulation and reduce systemic exposure, their efficacy can be limited by heterogeneous target receptor expression across tumors and patient populations, potentially leading to variable therapeutic outcomes.

A critical advantage of nanotherapeutic platforms lies in their ability to achieve site-specific drug delivery, which enhances therapeutic efficacy while minimizing off-target toxicity. Flynn et al. demonstrated this principle by developing anti-PSMA antibody-coated poly-L-lysine-grafted-polyethylene glycol (PLL-g-PEG)/GOX complexes that selectively targeted PMSA-expressing LNCaP prostate cancer cells, inducing pronounced cytotoxicity through localized ROS generation compared to untargeted GOX [68]. Similarly, hyaluronic acid (HA)-functionalized NPs enabled receptor-mediated uptake in CD44-overexpressing HeLa cells, triggering apoptosis via ROS accumulation and mitochondrial dysfunction [69].

Recent advances have further refined GOX delivery through innovative biomimetic and stimuli-responsive systems. Baiyan et al. engineered macrophage membrane-camouflaged mesoporous bioactive glass NPs (GOX-MBG) that evaded immune clearance while effectively delivering GOX to breast tumors, where it induced oxidative cell death [70]. Zhao et al. developed a near-infra red (NIR)-responsive covalent organic framework (COF) for spatiotemporally controlled GOX release [58], while Huo et al. designed an in-situ assembled GOX-poly(N,N'-dimethylamino-2-ethyl methacrylate) (PDMA) conjugate that improved stability, tumor retention, and safety through regulated H_2_O_2_ production [71]. Chen et al. leveraged the pH-sensitive degradation of zeolitic imidazolate framework-8 (ZIF-8) to create a tumor-selective GOX-delivery system, where acidic conditions and enzymatic byproducts triggered localized release, enhancing cytotoxicity while reducing systemic exposure [57]. These targeted strategies collectively address key challenges in GOX therapy by improving tumor accumulation, prolonging circulation, and minimizing off-target effects, thereby maximizing the therapeutic index of metabolic disruption combined with oxidative stress.

**Fig. 2:**
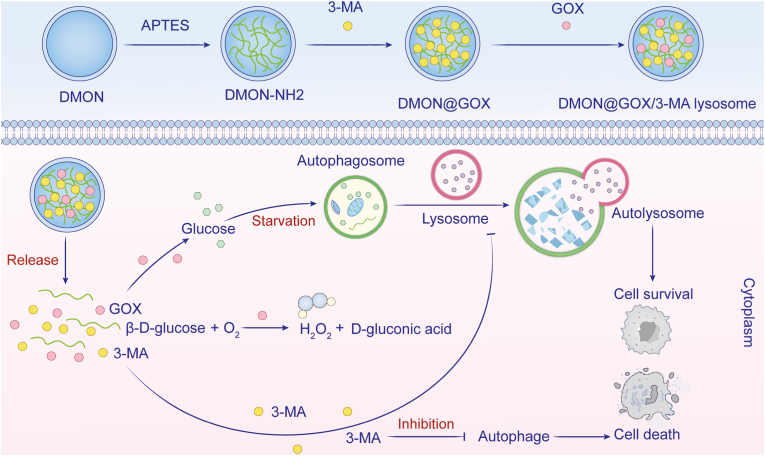
Schematic representation of dual GOX starvation and autophagy inhibition therapy. The autophagy blocker 3-MA was loaded into DMONs along with GOX for enhanced tumor starvation. Adapted from Ref. 58. **Abbreviations:** GOX, glucose oxidase; 3-MA, 3-methyladenine; DMONs, dendritic mesoporous organosilicon nanoparticles.

Despite its therapeutic potential, GOX-mediated monotherapy faces several biological and technical limitations that hinder clinical translation. The inherently hypoxic TME [1,14] and metabolic heterogeneity among cancer cell populations [72] significantly constrain therapeutic efficacy. Tumor cells demonstrate remarkable metabolic plasticity, adapting to glucose deprivation by utilizing alternative energy sources (lactate, amino acids, and fatty acids) [[53], [54], [55]] and activating pro-survival autophagy [52]. This adaptive response suggests that combining GOX with autophagy inhibitors like 3-methyladenine (3-MA) could enhance therapeutic outcomes, as demonstrated by Wu et al. using dendritic mesoporous organosilicon NPs (DMONs) for co-delivery (DMON@GOX/3-MA), which achieved superior tumor suppression compared to GOX alone (Fig. 2) [56].

The oxygen-dependent nature of GOX catalysis presents another key challenge, as enzymatic glucose oxidation exacerbates tumor hypoxia, ultimately limiting its own activity [24,28]. This paradox has been addressed by integrating oxygen-generating metal catalysts (Co, Cu, Fe, Mn, Ni) that convert GOX-produced H_2_O_2_ into oxygen via catalase-like activity [73,74]. Such combinatorial systems have been successfully incorporated into various nanoplatforms, including MOFs and metal oxide NPs, and noble metal NPs among others, have been developed that integrate metal catalyst function with the ability to load and release GOX in a controlled manner. including metal-organic frameworks (MOFs) and metal oxide NPs [24,28]. However, the resultant H_2_O_2_ overproduction risks off-target oxidative damage prompting innovative solutions Shan et al. bilirubin-loaded nanosystem that selectively scavenges excess H_2_O_2_ in normal tissues while maintaining antitumor efficacy [60]. Additional limitations include: (1) continuous glucose supply tumor vasculature, potentially overcome by combining anti-angiogenic agents [31,41], and (2) metabolic reprogramming via LOXL2-mediated glycolytic enzyme activation, suggesting therapeutic potential in dual GOX/LOXL2 inhibition [75]. These challenges have driven the field toward developing sophisticated combination therapies that leverage GOX’s catalytic activity while addressing its limitations through metabolic pathway co-targeting, oxygen self-supplying systems, redox-balancing approaches, and vascular normalization strategies.

### 3.1 Oxygen self-supplying systems

To overcome the oxygen-dependence paradox of GOX, a key strategy involves the co-delivery of oxygen-generating catalysts. Metal-based nanoparticles, such as MnO_2_ and CoFe, are designed to catalyze the decomposition of GOX-generated H_2_O_2_ into oxygen and water, mimicking catalase activity (2H_2_O_2_ → O_2_ + 2H_2_O). This approach not only replenishes local oxygen supply to sustain GOX catalysis but also releases metal ions (e.g., Mn^2+^, Fe^2+^) that participate in Fenton-like reactions, establishing a self-amplifying therapeutic cycle that enhances overall antitumor efficacy [44,60,73].

### 3.2 Metabolic pathway co-targeting

To overcome cancer cells’ metabolic plasticity, GOX is strategically combined with inhibitors targeting compensatory survival pathways. For example, co-encapsulation of autophagy inhibitors like 3-methyladenine (3-MA) or chloroquine prevents tumor cells from recycling intracellular components for energy, thereby promoting apoptosis [74]. Likewise, combining GOX with drugs like metformin, which inhibits mitochondrial complex I, simultaneously disrupts both glycolysis and oxidative phosphorylation pathways, inducing a catastrophic energy deficit that enhances tumor cell death [12].

### 3.3 Redox-balancing and targeted activation

To reduce off-target toxicity, GOX-based nanosystems are engineered for tumor-specific activation. pH-sensitive carriers, such as ZIF-8, remain stable during circulation but degrade in the acidic TME to release GOX locally [57]. Additionally, protective components like bilirubin can excess H_2_O_2_ in healthy tissues with neutral pH, while this protective function is suppressed in the acidic TME, establishing a selective therapeutic window that maximizes tumor-specific efficacy [60].

### 3.4 Vascular normalization strategies

GOX-based nanotherapeutics can be combined with anti-angiogenic agents to disrupt the continuous glucose supply to tumors. Rather than inducing extreme hypoxia, this strategy "normalizes" the aberrant tumor vasculature, improving nanocarrier delivery and reducing glucose influx, thereby amplifying the therapeutic starvation effect [31,62]. GOX-based nanotherapeutics can be combined with anti-angiogenic agents to disrupt the continuous glucose supply to tumors.

The following sections explore implementation of these practical strategies in synergistic combination therapies in detail. A summary of the key nanocarrier platforms developed for GOX-delivery, highlighting their distinct components, features, and primary mechanisms, is provided in Table 1.

**Table 1:** Representative nanocarrier platforms for for targeted delivery of glucose oxidase (GOX).

Nanocarrier Type	Key Components	Key Features/Mechanism	Primary Therapeutic Outcome	Ref.
**Polymeric Nanoparticles**	GOX, Chitosan/Alginate, PLGA, Dendrimers	Biocompatibility, sustained release, ease of functionalization.	Starvation therapy, localized oxidative damage.	[32,63]
**Metal-Organic Frameworks (MOFs)**	GOX, ZIF-8, Zr-MOF, Fe-MOF	High loading capacity, pH-responsive degradation, intrinsic catalytic activity.	Controlled release, synergistic starvation & chemodynamic therapy (CDT).	[46,47,57]
**Liposomes/Vesicles**	GOX, Lipid bilayer, Hemin, Fc	High biocompatibility, co-delivery of hydrophobic/hydrophilic agents.	Starvation therapy, enhanced CDT via embedded catalysts.	[30,87,112]
**Biomimetic Nanocarriers**	GOX, Cell membranes (e.g., macrophage, RBC), Viral capsids	Long circulation, immune evasion, inherent targeting.	Improved tumor accumulation, reduced systemic toxicity.	[59,66,70]
**Polymeric Nanoparticles**	GOX, Chitosan/Alginate, PLGA, Dendrimers	Biocompatibility, sustained release, ease of functionalization.	Starvation therapy, localized oxidative damage.	[32,63]

## 4 GOX-facilitated chemotherapy

Chemotherapy remains a cornerstone treatment for numerous malignancies, efficacy in chemosensitive tumors while serving as a critical adjuvant to radiotherapy regimens [76]. However, its clinical utility is frequently limited by the development of drug resistance and disease recurrence [77], compounded by the inherent nonspecific cytotoxicity that causes severe effects [78]. Emerging evidence indicates that metabolic starvation can potentiate chemo sensitivity [79,80], prompting the development of combination therapies integrating chemotherapeutic agents with glucose-depriving strategies [81,82]. Recent advances in nanomedicine have enabled the rational design of integrated platforms combining GOX with conventional chemotherapeutics. These systems leverage GOX's unique catalytic activity to achieve dual therapeutic benefits: (1) tumor-selective drug release through pH-responsive mechanisms, and (2) reversal of multi-drug resistance (MDR) via ATP depletion-mediated inhibition of efflux pumps [83,84].

Doxorubicin (DOX) remains a widely used chemotherapeutic for breast cancer, glioblastoma, and lung cancer, though its clinical utility is limited by dose-dependent cardiotoxicity cardiotoxicity and frequent development of resistance [85]. Nanocarrier encapsulation (micelles, liposomes, polymeric vesicles) has emerged as an effective strategy to improve DOX's tumor-specificity while reducing systemic toxicity [86]. Recent innovations have combined DOX with GOX to create synergistic therapeutic systems. Recently, Qi et al. developed pH-responsive hollow mesoporous silica NPs (HMSNs) co-loaded with GOX and DOX [84]. The acidic TME generated by GOX-mediated glucose oxidation triggered HMSN degradation and localized DOX release enhancing tumor-selective cytotoxicity. Xu et al. engineered a Cu^2+^-based MOF that simultaneously delivered GOX and DOX [46]. Within tumor cells, endogenous glutathione reduced Cu^2+^ to Cu^+^, which converted GOX-generated H_2_O_2_ into cytotoxic ·OH through Fenton reactions. This redox modulation not only induced oxidative damage but also significantly potentiated DOX activity against MDR lung cancer cells. This approach cleverly utilizes the tumor's own redox characteristics for specific activation; however, the variable concentration of GSH between different tumors could lead to inconsistent Fenton reaction efficiency and treatment response.

Chen et al. demonstrated another combinatorial approach using using ferrocene-modified nanovesicles (GOX.CP@FcNV) co-delivering GOX and cisplatin (CP) [87]. The system exploited three synergistic mechanisms: (1) glucose depletion via GOX catalysis, (2) Fe^2+^-mediated ·OH generation from H_2_O_2_, and (3) platinum-induced DNA damage. Notably, this combination therapy downregulated P-glycoprotein expression [88], overcoming a key resistance mechanism in chemorefractory tumors. Compared to cisplatin alone (CP@FcNV), the GOX-containing formulation showed superior efficacy *in vivo*, highlighting the potential of metabolic modulation to enhance conventional chemotherapy. Despite this promising synergy, the potential for ferrocence-derived toxicity and the complex pharmacokinetics of such multi-component systems require thorough long-term safety evaluation before clinical translation. These advanced delivery platforms address multiple challenges simultaneously: (1) improved drug solubility and stability, (2) tumor-specific release mechanisms, and (3) overcoming MDR. Reduced off-target toxicity while leveraging GOX's ability to remodel the TME for enhanced chemotherapeutic efficacy. The oxygen-depleting action of GOX creates an ideal microenvironment for activating HAPs, enabling targeted tumor therapy through precise spatiotemporal control. This approach capitalizes on GOX's ability to intensify hypoxic conditions in the TME, thereby selectively activating bioreductive compounds while sparing normoxic healthy tissues. Two prominent HAPs have shown promise in combination with GOX.

### 4.1 Tirapazamine (TPZ)

This benzotriazine-di-N-oxide compound undergoes enzymatic reduction in hypoxic conditions, generating cytotoxic free radicals that cause DNA strand breaks [89]. Zhou et al. demonstrated enhanced TPZ activation through GOX co-delivery in injectable alginate hydrogels. The -mediated oxygen depletion created localized hypoxia that triggered TPZ conversion, resulting in potent chemo-oxidative melanoma cell death [90].

### 4.2 Banoxantrone dihydrochloride (AQ4N)

**Fig. 3:**
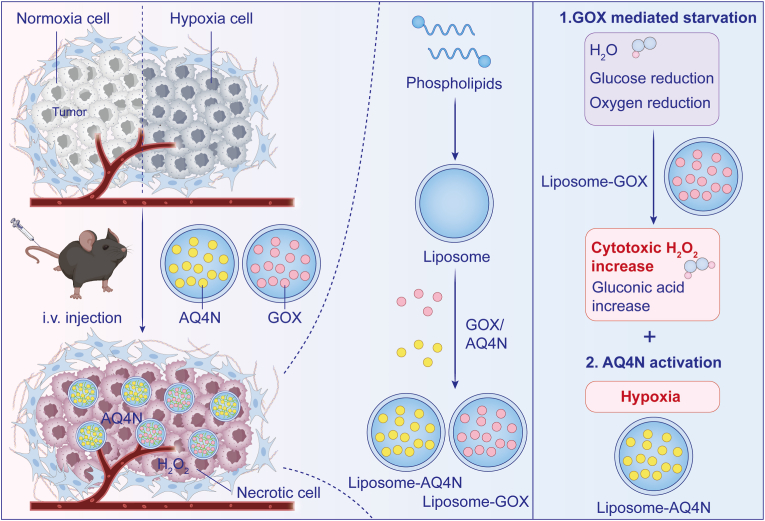
Schematic representation of synergistic starvation therapy and chemotherapy using GOX and the hypoxia-activated drug AQ4N. GOX-mediated depletion of oxygen led to hypoxia-responsive activation of AQ4N, resulting in selective tumor inhibition without affecting the well-oxygenated normal tissues. Adapted from Ref. 81. **Abbreviations:** GOX, glucose oxidase; AQN4, banoxantrone dihydrochloride.

As a topoisomerase II inhibitor activated by heme-containing reductases under hypoxia [91], AQ4N benefits significantly from GOX-induced oxygen starvation. Zhang et al. developed stealth liposomes co-encapsulating GOX and AQ4N that achieved: tumor-specific accumulation, dual glucose and oxygen depletion, hypoxia-selective AQ4N activation, and complete sparing of normal tissues (Fig. 3) [92].

These combinatorial approaches demonstrate three key advantages: (1) enhanced therapeutic specificity: Activation restricted to hypoxic tumor regions, (2) synergistic efficacy: Combined metabolic starvation and DNA damage, and (3) reduced systemic toxicity: Minimal impact on well-oxygenated tissues. The success of these systems highlights the potential of GOX to potentiate hypoxia-dependent therapies while addressing the challenge of insufficient endogenous hypoxia in many tumors. By artificially creating and maintaining therapeutic hypoxia through continuous oxygen consumption, GOX-based platforms may expand the clinical applicability of HAPs beyond traditionally hypoxic tumors.

The therapeutic potential of GOX extends beyond conventional chemotherapy through synergistic combinations with various anticancer compounds. A polymer-based nanoplatform combining GOX with curcumin, a natural polyphenol from turmeric with established antitumor properties [93], demonstrated enhanced cytotoxicity against breast cancer cells compared to free curcuminThe nanocomposite exerted its effects through increased ROS generation and mitochondrial membrane depolarization [94]. Another innovative approach combined GOX with metformin, an anti-diabetic drug that inhibits hexokinase 2 in the glycolytic pathway [95]. Meng et al. developed Arg-Gly-Asp (RGD) peptide coated, histidine-modified zeolitic imidazolate framework NPs (Met/GOx@His/ZIF-8∼RGD) that released both agents in a pH-responsive manner. While metformin blocked glycolysis and reduced ATP production, GOX depleted glucose stores, resulting in superior tumor suppression compared to monotherapies [12]. Researchers have also paired GOX and mitoxantrone (MTO), a topoisomerase inhibitor used for hematological malignancies. This combination platform achieved potent anti-tumor effects by merging GOX-mediated starvation/oxidative stress with MTO-induced DNA damage [96]. These studies collectively demonstrate GOX’s ability to enhance diverse therapeutic mechanisms when strategically combined with other antineoplastic agents.

## 5 GOX-facilitated oxidative therapies

Cancer cells exhibit a delicate balance between ROS production and antioxidant defenses, with metabolic reprogramming creating inherent oxidative stress [97,98]. While moderate ROS levels can paradoxically promote cancer cell survival by activating oncogenic pathways and suppressing tumor suppressor genes [97], tumor cells adapt to chronic oxidative stress by upregulating antioxidant systems, particularly through nuclear factor erythroid 2-related factor 2 (Nrf2) mediated transcriptional control [99]. However, when ROS accumulation exceeds cellular antioxidant capacity, it triggers various forms of cell death including apoptosis, ferroptosis, and necrosis [100,101].

Oxidative therapies exploit this vulnerability by generating cytotoxic free radicals, (·OH, O_2_^−^, and ^1^O_2_ that induce oxidative damage to cellular components, including proteins, nucleic acids, and membrane lipids, ultimately leading to genetic lesions, cell membrane disruption, and cell death [[101], [102], [103], [104]]. GOX significantly enhances these therapies by providing a continuous supply of H_2_O_2_, which serves as a substrate for metal-catalyzed Fenton and Fenton-like reactions. These reactions not only produce highly destructive hydroxyl radicals but also regenerate oxygen to sustain GOX activity, creating a self-amplifying therapeutic cycle [24,28,73,74]. The synergy between GOX and metal catalysts has been leveraged in multiple oxidative therapies. This section examines recent advances in nanoplatforms designed to optimize GOX-facilitated oxidative therapies, focusing on their mechanisms and therapeutic efficacy against resistant tumors.

### 5.1 Chemodynamic therapy (CDT)

CDT utilizes metal ions to generate cytotoxic ·OH from H_2_O_2_ through Fenton and Fenton-like reactions in tumor tissues [105,106]. The classical Fenton reaction utilizes ferrous ions (Fe^2+^) to convert H_2_O_2_ into ·OH (H_2_O_2_ + Fe^2+^ → Fe^3+^ + ·OH + OH^−^) [106], while Fenton-like reactions involve other metal ions (Cu^2+^, Mn^2+^, Co^2+^) or non-metal catalysts to facilitate similar redox. Effective CDT implementation faces several challenges, including insufficient endogenous H_2_O_2_ levels in tumors [107], and suboptimal pH conditions in TME (pH = 5–7) compared to the ideal Fenton reaction range (pH 2–4) [108]. Additionally, high levels of GSH concentrations in tumor tissues can inhibit Fenton reactions by chelating metal ions and scavenging the ·OH [109]. GOX addresses these limitations by continuously generating H_2_O_2_ through glucose oxidation while simultaneously acidifying the TME via gluconic acid production, thereby creating favorable conditions for metal-catalyzed ·OH generation [28]. Recent advancements have developed self-sustaining nanoreactors that combine GOX with metal catalysts, where GOX maintains H_2_O_2_ supply for Fenton reactions while the metal components perform dual functions: (1) producing ·OH through Fenton chemistry, and (2) generating oxygen via catalase-like activity to sustain GOX function [24,28,109]. These systems establish a synergistic cycle of glucose depletion, H_2_O_2_ production, metal-catalyzed ·OH generation, and oxygen regeneration, significantly enhancing therapeutic efficacy against tumors.

**Fig. 4:**
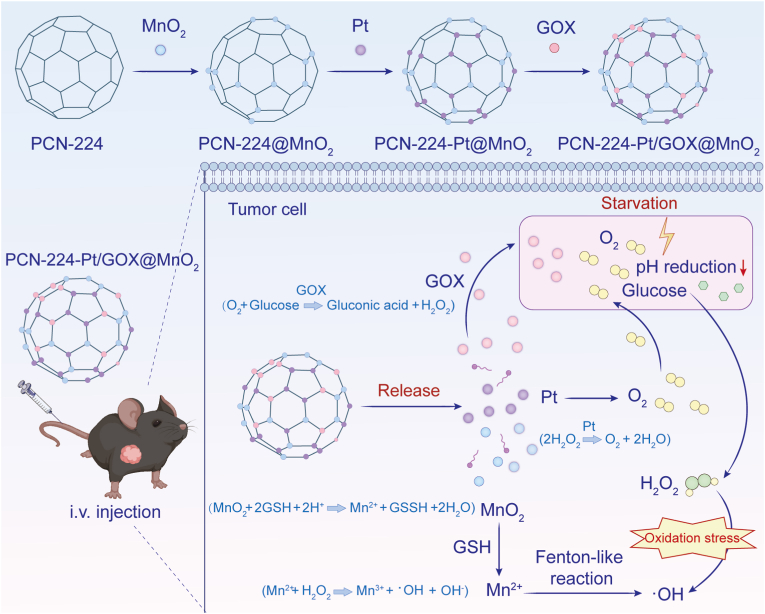
Schematic illustration of GOX-facilitated chemodynamic therapy. MnO_2_-coated NPs were loaded with GOX and CP for bimodal starvation/CDT. The CP/GOX@MnO2 NPs consumed GSH and generated high levels of ·OH and oxygen through the decomposition of H_2_O_2_. Adapted from Ref.100. **Abbreviations:** GOX, glucose oxidase, MnO_2_, manganese dioxide; NPs, nanoparticles; CP – cisplatin; GSH, glutathione; ·OH, hydroxyl radicals, H_2_O_2_, hydrogen peroxide.

Recent studies have demonstrated innovative approaches to combine GOX with metal catalysts for improved CDT. Ming et al. developed a pH-responsive Pd@Pt-GOX nanoconjugates that exhibited selective cytotoxicity against tumor cells both in vitro and *in vivo* while sparing normal cells, highlighting their potential for targeted therapy [110]. Zhu et al. engineered manganese oxide (MnO_2_)-coated NPs (Pt/GOX@MnO_2_) that simultaneously addressed multiple therapeutic challenges: these nanoconjugates effectively depleted GSH while generating substantial amounts of ·OH and oxygen through H_2_O_2_ decomposition in simulated TME conditions (Fig. 4) [44]. The Pt/GOX@MnO_2_ platform demonstrated significant efficacy against breast cancer cells, reducing viability in vitro and suppressing tumor growth *in vivo* [44]. This design successfully integrates multiple catalytic functions into a single platform; however, the potential immunogenicity of noble metal components (Pt) and the incomplete understanding of manganese ion clearance pathways present significant translational hurdles that need addressing.

Researchers have explored various other nanocarrier systems to optimize GOX-mediated CDT. MOF-NPs incorporating hafnium/manganese -porphyrin [47] or cobalt -ferrocene [111] complexes, along with iron hydroxide -loaded liposomes [112], have all shown promising anti-cancer effects through synergistic GOX-Fenton agent cascades. Notably, non-metal catalysts like hemin (an Fe^3+^-protoporphyrin complex) have also been employed [113]; Jiang et al. demonstrated that hemin/GOX-loaded Pluronic F127 micelles exhibited strong peroxidase-like activity in acidic TME conditions, leading to significant tumor growth inhibition in melanoma model [114]. The use of commercially available Pluronic F127 offers advantages for scalability and biocompatibility; however, the relatively weak structural stability of micellar systems may lead to premature drug release during circulation, potentially reducing tumor-specific delivery.

Another innovative approach utilized hemoglobin-loaded ZIF-8 NPs, where the heme-derived Fe^2+^ ions catalyzed the conversion of both endogenous and GOX-produced H_2_O_2_ into cytotoxic ·OH under acidic, showing potent activity across multiple cancer cell lines^106^. These diverse nanoplatforms share common therapeutic advantages: (1) enhanced tumor selectivity through responsive activation, (2) self-sustaining cycles of ROS generation and oxygen production, (3) dual metabolic and oxidative stress induction, and (4) improved pharmacokinetics and reduced off-target effects. The continued development of such integrated systems represents a promising direction for overcoming current limitations in cancer therapy, particularly for resistant and aggressive tumor types. Ferroptosis, an iron-dependent cell death pathway characterized by lipid peroxidation, is initiated through Fe^2+^-catalyzed Fenton reaction that generate ·OH [115,116]. Xu et al. developed a GOX-based nanoreactor loaded with iron phosphate (FePO) that synergistically CDT and ferroptosis. In this system, GOX-produced H_2_O_2_ and gluconic acid facilitated Fe^3+^ release from FePO, which was subsequently reduced to Fe^2+^ by tumor associated GSH. The Fe^2+^ then reacted with H_2_O_2_ via Fenton chemistry to produce cytotoxic ·OH that induced lipid peroxidation and ferroptotic cell death [117].

Metal-based NPs (Ag, Zn, Au) exhibit inherent anticancer properties through oxidative stress induction and apoptosis [118,119]. When combined with GOX in nanosystems, their therapeutic efficacy is significantly enhanced. Sun et al. demonstrated this using ZIF-8 MOFs to co-deliver GOX and AgNPs [120]. The acidic TME triggered ZIF-8 degradation, releasing Zn^2+^ and Ag ^+^ ions while GOX simultaneously starved tumor cells of glucose and generated H_2_O_2_. The Zn^2+^ ions provided additional therapeutic benefit by inhibiting glycolysis through glyceraldehyde-3-phosphate dehydrogenase (GAPDH) [120,121]. These studies demonstrate how GOX can potentiate both ferroptosis and metal ion therapies through controlled release mechanisms, metabolic disruption, and oxidative stress amplification.

### 5.2 Photodynamic therapy (PDT)

PDT represents a non-invasive treatment approach that generates cytotoxic ROS through light-activated photosensitizers [122]. While early PDT applications utilized visible light, current strategies increasingly employ near-infra red (NIR) light, particularly in the NIR-II window (1000–1700 nm), which offers deeper tissue penetration and reduced phototoxicity for treating solid tumors [123]. Modern photosensitizers span diverse categories, including organic compounds (porphyrins, phthalocyanines and indocyanines), noble metals, transition metal oxides, and upconversion nanoparticles (UCNPs) [124,125]. The photochemical mechanism involves photosensitizer excitation to a triplet state upon light absorption, which then generates ROS either through direct electron transfer (producing ·OH and O_2_^−^) or energy transfer to molecular oxygen (yielding ^1^O_2_^)^ [123,126]. Critical limitation remains oxygen dependence, as the hypoxic TME frequently leads to treatment resistance [127,128]. To address this, researchers have developed innovative nanoplatforms incorporating oxygen-generating components such as MnO_2_ and catalase [24,129,130]. More recently, cascade bioreactors combining GOX with metal catalysts have demonstrated enhanced efficacy by simultaneously: (1) improving oxygen availability through H_2_O_2_ decomposition, (2) depleting GSH to reduce antioxidant defenses, and (3) maintaining elevated H_2_O_2_ levels to support Fenton reaction [45,131,132]. These integrated systems successfully combine metabolic starvation, chemodynamic therapy, and photodynamic therapy into a unified therapeutic platform.

Zhang et al. developed an acid-responsive ZIF-8 nanoreactor co-loaded with GOX, chlorin e6 (Ce6), and MnO_2_ for enhanced PDT [45]. In this system, GOX-generated H_2_O_2_ was catalytically decomposed by MnO_2_ to produce oxygen, which both improved Ce6-mediated PDT efficacy upon light irradiation, and sustained GOX activity. The concurrent reduction of MnO_2_ by GSH yielded Mn^2+^ ions that functioned as a Fenton-like catalysts, enabling combined CDT. This integrated approach demonstrated potent antitumor activity against 4T1 tumors in mice, achieving near-complete tumor regression in irradiated subjects [45]. While this self-sustaining oxygen cycle elegantly overcomes the fundamental limitation of PDT, the treatment efficacy remains dependent on sufficient light penetration, which can be challenging for deep-seated or internal tumors, limiting its broad clinical application.

**Fig. 5:**
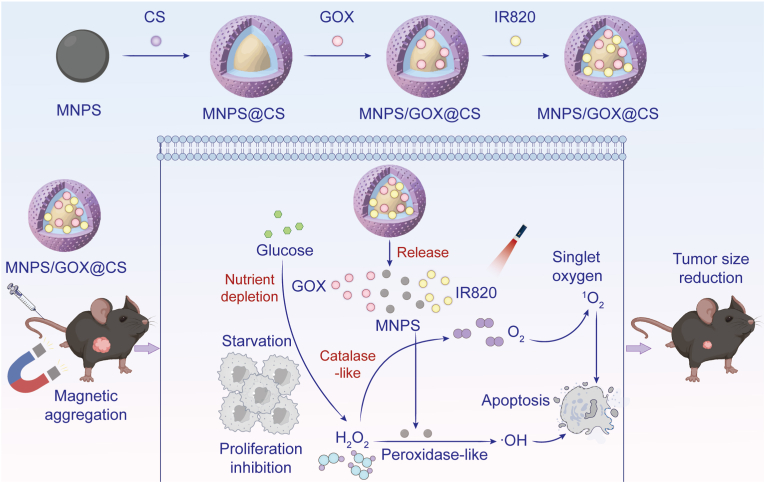
Schematic illustration of GOX-facilitated photodynamic therapy. GOX, Fe_3_O_4_ and IR820 were incorporated in a multifunctional nanosystem for synergistic CDT/PDT/starvation therapy. The cyclic production/consumption of H_2_O_2_ and oxygen by GOX and Fe_3_O_4_ promoted Fe^2+^-driven CDT and generated oxygen for IR820-induced PDT. Adapted from Ref. 126. **Abbreviations:** GOX, glucose oxidase; Fe_3_O_4_, ferric oxide; IR820, indocyanine green; CDT, chemodynamic therapy; PDT, photodynamic therapy; H_2_O_2_, hydrogen peroxide.

Jiale et al. created a theranostic nanosystem incorporating GOX and Fe_3_O_4_ with IR820 (an indocyanine derivative) as the photosensitizer [133]. The platform operated through a self-sustaining cycle in the TME: GOX-generated H_2_O_2_ was utilized by Fe_3_O_4_ to produce oxygen (alleviating hypoxia for CDT) and Fe^2+^ (enabling CDT), while IR820 enabled fluorescence imaging and Fe_3_O_4_ provided magnetic resonance imaging contrast (Fig. 5) [129,133].

UCNPs composed of lanthanide/actinide-doped ceramics, offer unique capabilities for PDT by converting penetrating near-infrared (NIR) light (980 nm) to higher-energy visible light (470 nm) [134,135]. Wang et al. engineered cancer cell membrane-coated micelles containing GOX and UCNPs [136]. Under NIR irradiation, the UCNPs emitted blue light that activated H_2_O_2_ (produced by GOX) to generate cytotoxic ·OH, while simultaneously enabling glucose depletion. This combined approach showed enhanced tumor growth inhibition compared to monotherapies, particularly under NIR-irradiation [136].

### 5.3 Other oxidative therapies

Sonodynamic therapy (SDT) employs ultrasound pulses to activate sonosensitizers, which generate ROS in the presence of oxygen [137]. Various sonosensitizers have been explored, including organic compounds (porphyrins, phthalocyanines), noble metal NPs, and metal oxides [138,139]. When activated by ultrasound, these molecules transition to excited states and subsequently produce ^1^O_2_ upon returning to ground state [137]. The deep tissue penetration capability of ultrasound makes SDT particularly valuable for treating visceral tumors [140], and its efficacy can be significantly enhanced when combined with glucose starvation approaches [141,142].

Bao et al. developed an innovative SDT platform using erythrocyte membrane-coated porphyrinic MOFs (PCN-224) co-loaded with GOX and platinum NPs (Pt-NPs) [59]. This system demonstrated multiple therapeutic advantages: the erythrocyte membrane coating improved biocompatibility and prolonged tumor retention, while acidic TME triggered Pt-NPs release. The Pt-NPs then catalyzed GOX-generated H_2_O_2_ to produce oxygen, which both sustained GOX activity and enhanced porphyrin-mediated SDT upon ultrasound activation [59]. This synergistic combination of starvation therapy and oxygen-enhanced SDT represents a promising approach for treating deep-seated malignancies.

Electrodynamic therapy (EDT) is a minimally invasive approach that employs metal NPs to catalyze the decompose H_2_O_2_ into ·OH when activated by an oscillating electric field [143]. This process is particularly effective in tumor cells due to their elevated chloride ion concentrations, which further amplify ROS generation under electric stimulation [144,145]. Lu et al. developed a synergistic platform combining GOX with electrocatalytic Pt nanospheres for enhanced starvation/electrodynamic therapy [146]. Under electro-stimulation, the Pt nanospheres generated substantial ·OH while simultaneously producing oxygen to sustain GOX activity, thereby creating a self-reinforcing cycle of glucose depletion and oxidative damage. This dual-action system significantly improved therapeutic efficacy by maintaining continuous GOX-mediated starvation while enhancing EDT-driven tumor cell death [146]. The precise spatiotemporal control of gas release is a major advancement; nevertheless, achieving consistent dosing and ensuring complete containment of the gas precursors during circulation remain significant technical challenges for clinical development.

## 6 GOX-facilitated photothermal therapy (PTT)

Cancer exhibits greater vulnerability to hyperthermia compared to normal cells due to their distinct metabolic profile, rapid proliferation, and aberrant vasculature [147]. PTT exploits this differential sensitivity sensitivity by employing photothermal transducing agents (PTAs) that convert near-infrared (NIR-I and NIR-II) light into localized heat [148]. Diverse PTA classes have been developed, including organic dyes, polymer NPs, and nanostructures derived from noble metals, transition metals, and metal oxides [149,150]. When activated by NIR light, these agents elevate tumor temperature beyond 42 °C, inducing cancer cell apoptosis through multiple mechanisms: (1) plasma membrane disruption, (2) DNA damage, and (3) cytoskeletal breakdown [151,152]. The precise spatial control of heat generation enables selective tumor ablation while minimizing effects on surrounding healthy tissues [148].

Recent advances have developed integrated nanosystems combining GOX with PTAs for enhanced cancer therapy. These platforms operate through reciprocal enhancement: GOX-mediated catalysis improves photothermal conversion efficiency, while NIR-induced hyperthermia boosts enzymatic activity [[48], [49], [50], [51]]. Zou et al. engineered a GOX-cyanine (Cy7) conjugate where GOX protected the heptamethine Cy7 from photo-oxidation by oxygen depletion, significantly improving photothermal stability and therapeutic efficacy against tumors [153]. He et al. created MnO_2_ nanosheet-loaded GOX (MNS-GOX) systems that established a self-sustaining cycle: glucose oxidation generated H_2_O_2,_ which Mn^2+^ decomposed to produce oxygen, thereby maintaining GOX activity while enabling photoacoustic imaging -guided therapy [154].

Yang et al. developed strontium/copper silicate nanosheets (SrCuSi_4_O_10_/SCNs) functionalized with GOX therapy [155]. Under NIR-II irradiation, SCNs mediated: (1) photothermal ablation via heat generation, (2) enhanced GOX activity through thermal acceleration, (3) pH-dependent release of Sr^2+^/Cu^2+^ for CDT. Similar synergistic effects were achieved with transition metal carbide [156] and black phosphorous [157] nanocomposites [157], nanocomposites, where photothermal heating simultaneously potentiated GOX-mediated starvation and CDT. These systems demonstrate robust control of deep-seated tumors through PTT and hyperthermia-augmented glucose starvation. Similar synergistic effects were achieved with transition metal carbide [156] and black phosphorus [157] nanocomposites, where photothermal heating simultaneously potentiated GOX-mediated starvation and CDT. These systems demonstrate improved penetration and efficacy against deep-seated tumors through combined thermal and metabolic targeting.

The effectiveness of PTT is often limited by tumor cells' upregulation heat shock proteins (HSPs), which confer thermotolerance by repairing heat-damaged proteins and maintaining cellular homeostasis during hyperthermia [158,159]. Since HSP synthesis is ATP-dependent [160], glucose deprivation through GOX-mediated starvation therapy presents a viable approach to suppress HSP expression and enhance PTT efficacy. This principle has been successfully demonstrated in several nanotherapeutic platforms. Zhu et al. developed erythrocyte membrane-camouflaged MOF nanoparticles co-loaded with GOX and gold nanorods (AuNRs), where GOX-induced glucose depletion suppressed HSP production in colon tumors, thereby potentiating AuNR-mediated PTT [161]. Similarly, liposomes formulations combining GOX with the NIR dye DiR (1,1′-dioctadecyl-3,3,3′,3′-tetramethylindotricarbo cyanine iodide) showed enhanced PTT effects against 4T1 tumors through HSP downregulation, achieving superior tumor growth and metastasis inhibition compared to DiR alone [162]. Qi et al. further expanded this strategy by encapsulating both GOX and the photosensitizer IR780 in ZIF-8 NPs, creating a multimodal system where: (1) IR780 generated simultaneous ROS and heat under NIR irradiation, (2) GOX-mediated ATP depletion suppressed HSP90, and (3) the acidic TME triggered controlled release of both agents [163]. These approaches collectively demonstrate how metabolic intervention through GOX can overcome a key resistance mechanism in PTT while enabling synergistic combination therapies. Although ATP depletion effectively suppresses HSPs, this strategy could potentially affect normal cells with high energy demands in the tumor vicinity, raising concerns about collateral damage that warrants careful investigation.

## 7 GOX-facilitated immunotherapy

Immunotherapy activates the host immune system to recognize and eliminate cancer cells through various approaches, including cytokines, immune checkpoint inhibitors, oncolytic viruses, genetically engineered immune cells, and vaccines [164]. Despite demonstrating significant anti-tumor effects, these therapies present notable clinical challenges such immune-related adverse events, development of treatment resistance, and variable patient response rates that limit their widespread application [[165], [166], [167]]. A major contributing factor to these limitations is the immunosuppressive TME [204], where regulatory immune cells and inhibitory cytokines actively block antitumor immune responses [168]. This immunosuppressive network creates a significant barrier to treatment success, particularly in immunologically "cold" tumors that fail to respond to current immunotherapies. The complex interaction between cancer cells and their surrounding immune microenvironment highlights the critical need for innovative strategies that can effectively modify the TME to enhance therapeutic outcomes while maintaining an acceptable safety profile.

**Fig. 6:**
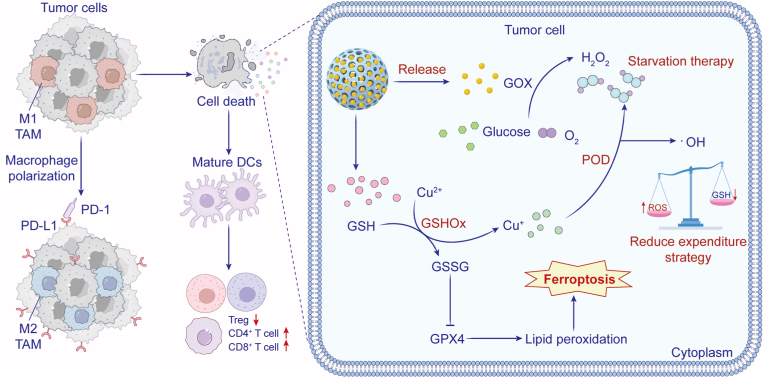
Schematic illustration of tumor starvation/chemodynamic/immunotherapy using GOX-linked Cu^2+^ nanosystem with peroxidase-like activity. H_2_O_2_ is oxidized to OH in the presence of Cu^2+^ and GSH levels via Fenton-like reaction. ROS elevation repolarized the M2 TAMs to the M1 phenotype, promoted maturation of DCs, and activated T cells. Adapted from Ref. 172. **Abbreviations:** GOX, glucose oxidase; Cu, copper; H_2_O_2_, hydrogen peroxide; GSH, glutathione; ROS, reactive oxygen species; TAMs, tumor-associated macrophages; DCs, dendritic cells.

The ROS can polarize generated through GOX-mediated therapy can transform the TME by converting tumor-associated macrophages (TAMs) from the immunosuppressive M2 phenotype to the tumoricidal M1 phenotype [169,170]. The ·OH produced by GOX and metal catalysts induce resulting in immunogenic cell death (ICD) in cancer cells, releasing tumor-associated antigens (TAAs) that antigen presenting cells to stimulate a T cell response [171,172]. Sun et al. demonstrated this effect using manganese -doped hollow mesoporous silica nanoparticles (HMSNs) to co-deliver AuNPs and GOX, which enhanced antitumor immunity through ICD [173]. Wang et al. developed a GOX-Cu^2+^ nanosystem where Fenton-like reactions converted GOX-generated H_2_O_2_ into ·OH, simultaneously increasing oxidative stress and reshaping the TME [61]. This approach: (1) shifted M2 TAMs to the M1, (2) matured dendritic cells, (3) increased cytotoxic T lymphocyte (CTL) infiltration while reducing regulatory T cells, and (4) boosted pro-inflammatory cytokines (IL-6, TNF-α), converting “cold” tumors to a “hot” state (Fig. 6) [61]. This represents a powerful strategy for remodeling the immunosuppressive TME; however, the potential for overstimulating the immune system and triggering cytokine release syndrome or autoimmune-like responses requires careful dose optimization and safety monitoring. Similar immune activation was achieved using Fe^3+^-GOX nanogel [174] and manganese-phthalocyanine NPs with GOX [175], both of which inhibited tumor growth by ROS-dependent TAM repolarization. These findings demonstrate GOX's dual role in directly killing tumor cells while creating an immune-favorable TME.

GOX-based nanotherapeutics have demonstrated significant potential in enhancing immune checkpoint blockades and cytokine therapies. The combination of Cu^2+^-GOX NPs and with anti-PD-L1(programmed death receptor ligand 1) antibodies showed remarkable efficacy in inhibiting both primary and distal tumors, where the ICD induced by Cu^2+^/GOX-mediated ROS production substantially augmented the checkpoint inhibitor's effects (Fig. 6) [61]. Similarly, Duan et al. reported that polymer-GOX conjugates combined with immune checkpoint inhibitors induced potent systemic anti-tumor immunity, effectively controlling distal tumor growth through ROS-mediated ICD [176]. The same research group further developed GOX-polycation-iron nanoconjugates that activated self-sustaining catalytic cascades to trigger ICD and produce abscopal effects significantly improving the outcomes of checkpoint blockade therapy [177].

Additional studies have explored membrane-camouflaged GOX systems for immunotherapy enhancement. Li et al. designed cancer cell membrane-coated GOX/hemin MOF NPs that utilized heme iron to convert H_2_O_2_ to ·OH, thereby promoting ICD and dendritic cell (DC) activation. When combined with anti-PD-L1 therapy, this platform significantly increased CD8^+^ CTL infiltration and tumor suppression [62]. Stinson et al. demonstrated that localized delivery of a GOX nanoformulation with interferon alpha induced durable tumor control in mouse models by enhancing tumor antigen presentation through dendritic cells activation [178]. These findings collectively establish that GOX-mediated catalytic therapy can potentiate immunotherapy by: (1) remodeling the immunosuppressive TME, (2) inducing ICD to enhance tumor immunogenicity, and (3) promoting antigen presentation and T cell activation. The ability of GOX to convert metabolic modulation into immune activation represents a promising strategy for overcoming resistance to current immunotherapies.

## 8 GOX-facilitated gas therapy

Gas therapy utilizes therapeutic gases including hydrogen (H) [179], carbon monoxide (CO) [180], nitric oxide (NO) [181], hydrogen sulfide (H_2_S) [182], and sulfur dioxide (SO_2_) [183,183] to target tumors through distinct mechanisms. While NO and CO directly induce tumor cell apoptosis, H and CO can mitigate therapy-induced inflammation [184]. These gases also potentiate conventional treatments chemotherapy and radiotherapy [184], their clinical application is limited by poor solubility and uncontrolled diffusion [185]. Recent advances have employed GOX-based systems to enable tumor-specific gas release through metabolic stimuli [186,187].

**Fig. 7:**
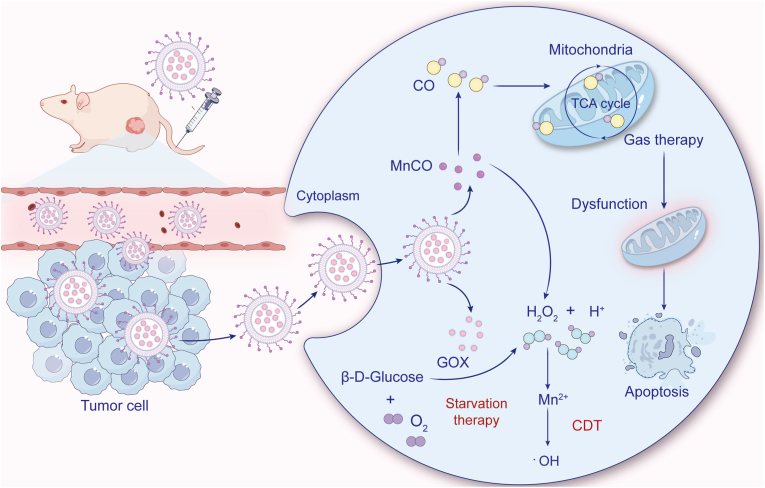
Schematic illustration of GOX-facilitated gas therapy using H_2_O_2_-responsive CO nanogenerator. The catalytic action of GOX depleted glucose, augmented in-situ CO release, and generated ·OH for CDT through Mn^2+^-mediated Fenton reaction. Adapted from Ref. 189. **Abbreviations:** GOX, glucose oxidase; H_2_O_2_, hydrogen peroxide; CO, carbon monoxide; CDT, chemodynamic therapy; ·OH, hydroxyl radical.

For CO delivery, Wu et al. developed hollow mesoporous organosilica NPs co-loaded with GOX and manganese carbonyl (MnCO) [188]. The acidic byproducts (gluconic acid and H_2_O_2_) of GOX-mediated glucose oxidation CO release from MnCO, simultaneously inducing apoptosis through respiratory chain inhibition while enhancing starvation therapy [189]. Wang et al. engineered a zirconium (Zr)-based MOF platform (GOX-MnCO@Zr) that coupled CO release with CDT [189]. Here, GOX actively both depleted glucose and generated H_2_O_2_ for Mn^2+^-catalyzed ·OH production, while the acidic TME promoted controlled CO release, achieving significant tumor suppression (Fig. 7) [189].

NO therapy presents unique challenges due to its concentration-dependent effects: while physiological NO levels promote vasodilation and antioxidant effects, elevated concentrations induce apoptosis/ferroptosis through reactive nitrogen species (RNS) like peroxynitrite (ONOO^−^) [190,191]. GOX-based systems address this by using L-arginine (L-Arg) as a substrate, GOX-generated H_2_O_2_ in acidic conditions drives localized NO production for synergistic starvation/gas therapy [187]. Similarly, SO_2_ donors have been combined with GOX to exploit its oxidative stress-enhancing properties [192]. Chu et al. designed Mg/Al nanosheets loaded with sulfite and GOX, where tumor acidosis triggered SO_2_ release that reacted with H_2_O_2_ to generate cytotoxic free radicals [193]. These approaches demonstrate how GOX-mediated metabolic modulation can enable precise spatiotemporal control of therapeutic gases while minimizing systemic toxicity.

## 9 Summary and outlook

GOX-mediated starvation therapy represents an innovative approach in cancer treatment by targeting the characteristically high glucose consumption of tumor cells. The development of advanced nanocarrier systems has largely overcome previous challenges associated with systemic GOX administration, including off-target toxicity, short circulatory half-life, immunogenicity, and poor *in vivo* stability [24,28,34]. Researchers have engineered various various GOX-based nanodrugs capable of tumor-specific catalytic therapy, demonstrating potent anticancer effects through both targeted glucose deprivation and ROS-induced oxidative damage. Importantly, the catalytic activity of GOX enhances multiple therapeutic modalities by dynamically modulating key TME characteristics - including acidity, H_2_O_2_ concentration, and oxygen levels - thereby improving outcomes when combined with chemotherapy, CDT, PDT, PTT, gas therapy, and immunotherapy [24,28]. In recent years, numerous sophisticated nanoplatforms have successfully implemented this cascade strategy for synergistic cancer therapy. This review systematically examines the progress in GOX-facilitated multimodal therapeutic approaches and their evolving role in oncology. The synergistic mechanisms and advantages of different GOX-facilitated therapy strategies are comparatively illustrated in Table 2, while a comprehensive overview of specific representative nanoplatforms is provided in Table 3.While glucose starvation shows promise, a significant limitation lies in cancer cells’ metabolic plasticity - their ability to adapt by utilizing alternative nutrient sources such as amino acids and fatty acids [55]. Moreover, the extensive tumor vasculature facilitates continuous glucose supply from circulation [24,74], while interactions with immune and stromal cells in the TME provide additional metabolic support [194]. These challenges may be addressed through combination therapies targeting multiple metabolic pathways simultaneously or dual inhibition of both glucose uptake and and utilization [24], though this requires deeper understanding of tumor metabolic networks. Importantly, GOX-based nanoreactors offer a compelling solution by enabling multimodal synergistic therapies. The catalytic production of H_2_O_2_ and gluconic acid creates favorable conditions for therapeutic reactions regardless of native TME characteristics. For example, metal catalysts can utilize the generated H_2_O_2_ in the Fenton reactions to produce cytotoxic free radicals. The resulting redox imbalance not only enhances tumor cell sensitivity to chemotherapy but also promotes immune activation through macrophage repolarization to the pro-inflammatory M1 phenotype. Additionally, GOX-induced acidification and hypoxia provide ideal conditions for controlled release of TME-responsive therapeutic agents [[33], [34], [35], [36],^39^].

**Table 2:** Overview of multimodal cancer therapy strategies facilitated by glucose oxidase (GOX).

Therapeutic Modality	Synergistic Partners with GOX	Key Synergistic Mechanism	Major Advantage	Ref.
**Chemotherapy**	Doxorubicin, Cisplatin, HAPs (e.g., AQ4N)	ATP depletion reverses drug efflux; TME acidification triggers drug release; GOX-induced hypoxia activates HAPs.	Overcomes multidrug resistance (MDR); enables tumor-selective drug activation.	[84,87,92]
**Chemodynamic Therapy (CDT)**	Fenton agents (Fe^2+^, Cu^2+^, Mn^2+^)	GOX provides sustained H_2_O_2_ supply; gluconic acid optimizes pH for Fenton reaction.	Self-supplying ROS generation; amplifies oxidative damage beyond starvation.	[44,46,117]
**Photodynamic Therapy (PDT)**	Photosensitizers (Ce6, IR820), O_2_ generators (MnO_2_)	GOX-induced hypoxia is alleviated by O_2_ from H_2_O_2_ decomposition; continuous O_2_ supply enhances ^1^O_2_ yield.	Breaks the hypoxia barrier of traditional PDT; self-sustaining catalytic cycle.	[45,133,136]
**Photothermal Therapy (PTT)**	Photothermal agents (AuNRs, Cy7, BP)	Hyperthermia increases GOX activity and blood flow; GOX-mediated ATP depletion inhibits protective HSPs.	Mutual enhancement of effects; overcomes thermoresistance for deeper ablation.	[153,161,163]
**Immunotherapy**	Immune checkpoint inhibitors (anti-PD-L1)	GOX/ROS induce immunogenic cell death (ICD) and repolarize M2 macrophages to M1 phenotype.	Converts "cold" tumors to "hot"; primes tumor for checkpoint blockade.	[61,62,176]

**Table 3:** Representative GOX-based nanoplatforms for multimodal cancer therapy: mechanisms, therapeutic modes, and tumor targets.

Specific Example (Nanoplatform)	Therapeutic Mode(s)	Tumor Metabolism Pathway Targeted	Special Advantages/Mechanism	Demonstrated Tumor Type (in study)	Ref.
**DMON@GOX/3-MA**	Starvation, Autophagy inhibition	Glycolysis, Autophagy	Blocks autophagy as a resistance pathway to glucose starvation	Not specified (in vitro/vivo model)	[56]
**GOX/Cu^2+^-MOF with DOX**	Chemotherapy (Doxorubicin), CDT	Glycolysis, Oxidative phosphorylation	GSH-activated Fenton reaction; reverses MDR via ATP depletion	Lung cancer	[46]
**GOX.CP@FcNV (Cisplatin)**	Chemotherapy (Cisplatin), Starvation, CDT	Glycolysis, DNA repair	Self-supplied H_2_O_2_ for Fe^2+^-mediated CDT; downregulates P-gp	Multidrug-resistant tumors	[87]
**Liposomes (GOX + AQ4N)**	Chemotherapy (Hypoxia-activated), Starvation	Glycolysis, Hypoxic signaling	Creates its own hypoxia for selective prodrug activation; spares normal tissue	Not specified (in vitro/vivo model)	[92]
**Pt/GOX@MnO_2_**	Starvation, CDT	Glycolysis, Antioxidant defense (GSH)	Depletes GSH; self-generates O_2_ from H_2_O_2_; multi-enzyme mimic	Breast cancer	[44]
**ZIF-8/GOX/Ce6/MnO_2_**	Starvation, CDT, PDT	Glycolysis, Hypoxic signaling	Self-replenishing O_2_ cycle for enhanced PDT; pH-responsive release	Breast cancer (4T1)	[45]
**GOX-Cy7 Conjugate**	Starvation, PTT	Glycolysis	O_2_ depletion protects cyanine from photobleaching, enhancing PTT stability	Not specified (in vitro/vivo model)	[153]
**GOX/AuNRs in MOF**	Starvation, PTT, Immunomodulation	Glycolysis, HSP synthesis	Glucose/ATP depletion inhibits HSPs, overcoming thermoresistance	Colon cancer	[161]
**GOX/Cu^2+^ Nanosystem**	Starvation, CDT, Immunotherapy	Glycolysis, Oxidative phosphorylation	Fenton-like reaction induces ICD and repolarizes TAMs (M2→M1)	Not specified (in vitro/vivo model)	[61]
**GOX-MnCO@Zr**	Starvation, CDT, Gas Therapy (CO)	Glycolysis, Mitochondrial respiration	H_2_O_2_ and acidity trigger tumor-specific CO release for synergistic apoptosis	Not specified (in vitro/vivo model)	[189]

Despite its potential, GOX-based nanocatalytic therapy remains in its early developmental stages, with several challenges hindering clinical translation. A primary concern is the unestablished long-term biosafety profile of these nanomedicines. Many current nanocarriers are non-biodegradable, raising concerns about potential systemic toxicity while certain metal catalysts may introduce additional safety risks [194].

A pivotal step towards clinical translation is rigorous preclinical evaluation of GOX-nanocarriers’ pharmacokinetics, biodistribution, and long-term safety in preclinical models. Encouragingly, recent studies have begun to address these gaps. For example, a biodegradable poly(γ-glutamic acid)-GOX-carbon dot nanoparticle system enabled real-time *in vivo* tracking of its biodistribution and catalytic activity, demonstrating high tumor accumulation and yielding invaluable pharmacokinetic data [195]. In parallel, the encapsulation of GOX within a ZIF-8 MOF has been shown to significantly improve its stability and circulation half-life compared to free enzyme, while enabling tumor-specific release under acidic TME [196]. On the safety front, engineered GOX-based nanoreactors in recent work achieved potent tumor inhibition without histopathological damage to major organs, and demonstrated effectient clearance to mitigate long-term toxicity [197]. Finally, design innovations such as self-supplying H_2_O_2_ systems enhance enhance therapeutic efficacy while minimizing systemic exposure, thereby improving the biosafety profile and therapeutic window of GOX-based therapies [198]. These studies collectively demonstrate that rational nanomaterial design must not only maximize therapeutic efficacy but also ensure a robust safety profile essential for clinical translation.

Comprehensive studies evaluating long-term toxicity, pharmacokinetics, and pharmacodynamics are urgently needed. Patient-derived xenografts could provide more clinically relevant safety and efficacy data compared to traditional cell line-derived models [29]. The complex multi-step synthesis of therapeutic nanocarriers presents another hurdle, with scalability challenges [28,29] and potential GOX structural alterations during conjugation processes [199]. To address these hurdles and steer the field toward clinical translation, future research should prioritize the following specific directions:

**Engineering Biodegradable and "Smart" Nanocarriers:** To address long-term toxicity and persistence within the body, designing fully biodegradable and stimuli-responsive nanocarriers is essential. Promising platforms include polymeric nanoparticles from materials such as poly(lactic-co-glycolic acid) or acetalated dextran as well as MOFs incorporating biodegradable linkers. These systems are engineered to degrade into non-toxic byproducts after completing their therapeutic function, enabling efficient body clearance and directly mitigating the core biosafety concerns associated with non-degradable nanomaterials [200].

**Harnessing Multi-Omics for Predictive Therapy:** To counteract metabolic plasticity, future therapeutic strategies should integrate multi-omics technologies - including transcriptomics, proteomics, metabolomics - to generate patient-specific metabolic signatures [206]. Such comprehensive profiling would enable the prediction of tumor adaptive responses to glucose starvation and the rational design of personalized combination regimens. For example, tumors exhibiting strong glutamine dependence could be treated with GOX in combination with a glutaminase inhibitor, whereas those favoring fatty acid oxidation could be co-targeted using GOX and etomoxir, thereby pre-emptively blocking alternative metabolic escape pathways.

**AI-Driven Design and In Silico Screening:** The inherent complexity of nanomaterial engineering can be effectively addressed using artificial intelligence (AI) and machine learning based computational models. These approaches can predict how nanocarrier properties - such as size, charge, and surface chemistry-interact with biological systems, enabling the optimization of formulations for maximal tumor accumulation and minimal off-target effects. By rapidly screening vast parameter spaces in silico, AI-driven platforms can significantly accelerate the development of safe and efficacious GOX nano-formulations, guiding the selection of the most promising candidates for experimental validation [201].

**Advanced Preclinical Models for Reliable Safety Assessment:** To obtain clinically meaningful insights into both efficacy and safety, it is essential to move beyond conventional cell line models and adopt patient-derived organoids and xenografts (PDOs/PDXs). These advanced systems more accurately recapitulate the heterogeneity and microenvironmental complexity of human tumors, generating data that better predict therapeutic responses and potential toxicities prior to clinical translation [29].

Importantly, the TME alterations induced by GOX - including intensified hypoxia, oxidative stress, and acidosis - may paradoxically facilitate tumor progression [43,202], underscoring the need for deeper investigation to inform rational combination therapies. Nevertheless, accumulating evidence supports GOX-based starvation therapy as a compelling anticancer modality, with recent advances in nanocarrier engineering offering a strong foundation for future innovation in multimodal cancer treatment.

## 10 Concluding remarks

The evolution of glucose oxidase (GOX) from a basic metabolic enzyme into a central catalyst of advanced nanotherapeutic platforms exemplifies the transformative potential of interdisciplinary scientific research and innovation. By exploiting the metabolic dependencies that underpin malignant growth, GOX-based nanotherapies convert a core cancer vulnerability into a powerful and flexible modality for multimodal intervention. Although significant translational challenges remain - including metabolic adaptability, pharmacokinetic complexity, and long-term biosafety - the progress outlined in this review defines a compelling trajectory for the field. Ultimately, future breakthroughs will emerge not from increasingly elaborate constructs, but from the development of smarter, safer, and more adaptive systems capable of engaging the tumor microenvironment with molecular precision. As efforts to strategically reprogram tumor metabolism accelerate, the prospect of GOX-enabled nanocatalysis advancing from preclinical promise to meaningful clinical impact appears not only plausible, but increasingly attainable.

## CRediT authorship contribution statement

**Bin Wang:** Writing – original draft, Investigation. **Naren Bao:** Writing – original draft, Investigation. **Limei Wang:** Writing – original draft, Investigation. **Jian Xiong:** Writing – original draft, Investigation. **Danyang Li:** Writing – original draft, Investigation. **Yutao Wang:** Writing – review & editing, Supervision, Conceptualization. **Ghulam Md Ashraf:** Writing – review & editing, Supervision, Conceptualization. **Xu Wang:** Writing – review & editing, Supervision, Conceptualization.

## Funding

None.

## Declaration of competing interest

The authors declare that they have no conflicts of interest.

## Data Availability

No data was used for the research described in the article.
